# Colonization Rates in a Metacommunity Altered by Competition

**DOI:** 10.1371/journal.pone.0088344

**Published:** 2014-02-13

**Authors:** Shajini Jeganmohan, Caroline Tucker, Marc W. Cadotte

**Affiliations:** 1 Department of Biological Sciences, University of Toronto-Scarborough, Toronto, Ontario, Canada; 2 Ecology and Evolutionary Biology, University of Toronto, Toronto, Ontario, Canada; McGill University, Canada

## Abstract

Competition and colonization are two mechanisms that are important for determining coexistence and species diversity in spatially structured habitats. However, these mechanisms may not be independent as species can exhibit behavioral or physiological changes in response to competition that alters their colonization rates. This study examines the effect of interspecific interactions on the colonization rates of four microscopic species (three ciliates and a rotifer) in aquatic microcosms. Two species showed significant reductions in the time to colonize patches when confronted with a competitor, one was a good disperser (*Colpidium striatum*) and the other was the slowest disperser (*Philodina spp.*). These results indicate that species’ colonization rates in a metacommunity can vary depending on the presence of competitors. Thus, we suggest that predictions based on heuristic tradeoffs between competition and colonization should consider effects of common biotic interactions such as competition.

## Introduction

Organisms in an environment face limitations of many kinds, including abiotic environmental conditions, resources, space, predation, and parasites or diseases. These limitations often require that organisms disperse from habitat to habitat, either away from a parent or highly populated areas [1]–[3], or when environmental parameters change, making some habitats unsuitable [4], [5]. Organisms that are able to arrive first at a new habitat may have an advantage, which depends on both the ability and rate of movement to the new habitat as well as establishment and reproductive success in the new site (collectively determining colonization rates of the new patch). This first component of colonization - the act of moving from one patch to another– is in turn dependent on a species’ intrinsic rate of increase (and so the number of individuals available for emigration) and the dispersal capacity of the species.

However, traits which tend to allow species to have high colonization rates, such as dispersal ability, may tradeoff with other traits by allocating resources away from growth, maintenance, or reproduction [6]. It has been suggested that species cannot maintain both high dispersal ability and high growth rates under intense competition, leading to tradeoffs in the allocation of energy to processes that influence dispersal and competition abilities [7], [8]. Such a tradeoff between dispersal or colonization rates and competitive success [9]–[12] has been used to explain how unequal competitors can coexist in homogeneous metacommunities if species that are poor competitors have traits that allow them to be superior colonizers, enabling them to quickly occupy patches where a superior competitor goes locally extinct [11], [13]–[16]. However, many traits may contribute to differences in colonization rates between species and dispersal ability may be influenced by other tradeoffs such as predation (Barraquand and Murrell 2012).

Most theoretical and empirical studies of colonization differences between species and coexistence assume that species have constant rates of colonization that do not change with context. However, dispersal is strongly dependent on species’ densities, and may also be affected by external stimuli or changes in resources or environmental conditions. Protist dispersal can change in relation to food abundance, temperature, and moisture gradients [17]. Migratory behaviors of many bird species including the black-throated blue warbler, *Dendroica caerulescens* [18], are driven by seasonal changes, while in response to herbivory, plant compensatory growth can result in higher seed production [19], and thus a higher net colonization. Hence colonization rate a species may achieve is determined both by intrinsic characteristics of a species and by environmental and biotic conditions, although the extent to which each of these factors can alter colonization rates is less explored.

We used protozoan microcosms to examine how the competitive environment influences colonization of empty patches in a metacommunity. The species used in this experiment (*Paramecium tetraurelia, Colpidium striatum, Blepharisma spp*., and *Philodina spp.*) have been shown to exhibit substantial differences in their rate of colonization of empty patches [10], which plays an important role in species coexistence and patch diversity in metacommunities [13]. These past results suggested *a priori* a tradeoff between the superior colonizing species (*Paramecium tetraurelia, Colpidium striatum*, which also tended to be small-bodied and have faster rates of dispersal) and the superior competitors (*Blepharisma spp*., and *Philodina spp.,* which tended to be larger-bodied species). However, we predicted that the magnitude of the colonization rate a species might achieve could be dependent on the biotic environment. We predicted that in our experiment the putative colonizer species (i.e., *Paramecium* and *Colpidium*) would be most likely to increase colonization into new patches when competitors were present. This increase in colonization rates could be through response to stimuli (such as reduced food availability) leading to plastic changes in morphology and physiology [20], altered dispersal patterns [21], [22], or a selective response for heightened dispersal in the presence of competition. If there is a strong tradeoff between movement rates and competitive success, it is possible that larger-bodied species (i.e., *Blepharisma*, and *Philodina*) are less able to increase their dispersal in high competition environments. Thus we predicted that colonization rates are reinforced by the presence of interspecific competition.

## Methods

**Figure 1:**
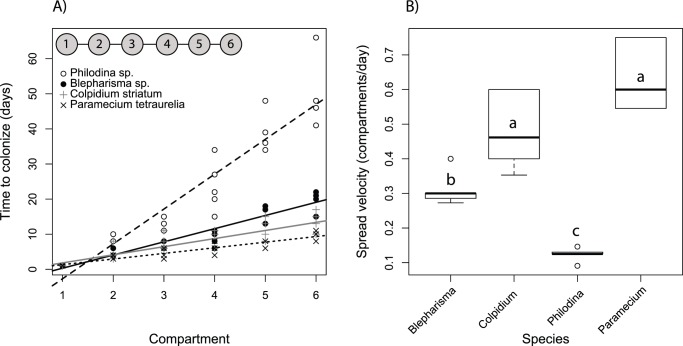
Species differ in how quickly they colonize patches. A) The time taken by each species to colonize the six patches within the single-species treatment. Lines fit by linear regression. Numbered circles at the top left of panel shows the experimental metacommunity. B) Species spread velocities (number of patches colonized per day). Letters indicate significant differences (P<0.05) among species.

We constructed protist microcosms using 100mL square Nalgene bottles (Nalg Nunc, Rochester, New York, USA), each of which served as discrete patches in the landscape. Each metacommunity was formed from six of these Nalgene bottles connected together in series with 5.5cm-long Nalgene tubing (Fig. 1). Nalgene tubing was connected between bottles using spouts fitted into openings drilled and threaded at the 50mL mark. The spouts and tube connections were sealed with silicone to prevent leaks and this entire apparatus was autoclaved prior to use. Each patch contained 100mL of protist media, made with protozoa pellets (Carolina Biological Supply, Burlington, NC) (0.46 g/L), vitamin, mineral, and amino acid supplement (Kent Marine, Franklin, WI)(218 uL/L), and sterilized wheat seeds (2 seeds/L). Protist media was autoclaved, inoculated with *Bacillus subtilis, Bacillus megaterium, Micrococcus roseus, Proteus vulgaris,* and *Staphylococcus epidermidis*, and incubated for at least two days to allow bacterial populations to reach carrying capacity.

Our microcosms included protist species *Paramecium tetraurelia*, *Colpidium striatum*, and *Blepharisma spp*., and the rotifer species *Philodina spp*., ordered from a biological supplier (Boreal Northwest, St. Catharines, Ontario, Canada). To examine how biotic interactions affect patch colonization, we established three experimental treatments. First, we measured the colonization rate into empty patches when a species was present as a monoculture in a single patch within the metacommunity. We used the first treatment to quantify species’ colonziation rates in the absence of interspecific competition. We added 500 individuals to the first patch and allowed them to disperse throughout the apparatus (Fig. 1). Enumerations were made three times a week; the day when protists were first added to a metacommunity was considered day one. We enumerated samples of 1mL from each patch in the metacommunity and replaced this volume with fresh media. Connecting tubes were blocked with clips to prevent backflows during sample removal. Sampling ended when at least one individual of the focal species was observed in each compartment.

The second treatment looked at rate of colonization when two species were competing: we selected species shown to be a good colonizer but poor competitor (*C. striatum)* and a one good competitor but poor colonizer (*Blepharisma spp.)* [13]. The two species were added sequentially: first 500 individuals of a species were added to the first compartment, and then following the first enumeration, 760 individuals of the remaining species were added to each compartment. Subsequent enumerations were carried out three times a week with replenishment of fresh media, as for the single-species assemblages. Replicates were ended when the original species reached the final compartment.

The final treatment included all four protist species. The mixed-species treatment was used to see colonization rates of each species differed when interspecific competition was highest in terms of the number of species competing. 500 individuals of each species were added simultaneously to the first patch. Replicates in the treatment were considered finished when at least one individual from all four species was observed in all patches of the metacommunity.

### Statistical Analyses

We calculated the colonization rate of each patch in the metacommunity in two ways for the monocultures. First, we used linear regression to relate time to patch colonized. Second, we used an ANOVA to determine if there were significant differences in colonization rate (number of patches colonized/day) among the species. Individual species rates were then compared using Tukey’s Honest Significant Differences test.

Next we determined if species had different colonization rates in the presence of the competitors. For each species, we determined the effect of the competition treatment on the amount of time required to arrive in the last patch in the metacommunity. We removed outliers as indicated by Studentized residuals. We used ANOVAs and Tukey’s Honest Significant Differences tests to determine treatment effects. All analyses were run using R 2.9.1 [23]. Not all treatments ended with five replicates since some metacommunities were removed due to contamination.

## Results

Species in the single-species treatments colonized all patches within 66 days (Fig. 1). The large-bodied rotifer *Philodina* took the longest time to colonize the entire metacommunity, up to 66 days, while the large bodied ciliate, *Blepharisma*, took approximately 20 days for complete colonization of metacommunity patches. *Colpidium and Paramecium*, both small-bodied ciliates, had the fastest times (Fig. 1).

**Figure 2:**
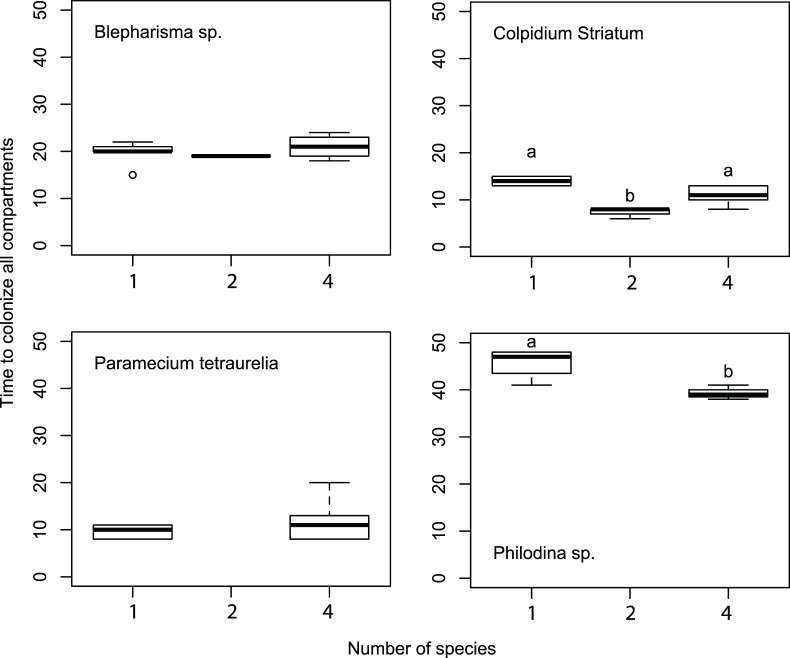
Box plots showing the time required for each species to colonize the last (sixth) patch. Richness treatments include the single species monocultures, two species (focal species plus one competitor) and four species (focal species plus three competitors) treatments. Letters indicate significant differences (P<0.05) among richness treatments.

Across the three treatments (and hence intensity of competitive interactions), our results showed species dependent effects of interspecific competition on colonization rates. *Blepharisma* and *Paramecium* did not show significant changes in colonization rates (Table 1, Fig. 2). In contrast, *Colpidium* and *Philodina* significantly increased their colonization rates (p = 0.0148 and p = 0.00194, respectively, Table 1, Fig. 2) in response to changes in the competitive environment. Tukey’s Honest Significant Differences tests indicated that *Colpidium* colonized empty patches most rapidly in the two-species treatment and that *Philodina* colonized empty patches more rapidly in the four-species treatment compared to when in monoculture (Fig. 2).

**Table 1:** ANOVA results for the comparison between competition treatment and the time taken for each species to arrive in the last patch.

Species	Effect	Df	SumSq	MeanSq	F	P-value
*Blepharisma spp.*	Competition	2	22.58	11.292	1.198	0.351
	Residuals	8	75.42	9.427		
*C. striatum*	Competition	2	67.86	33.93	6.326	0.0148
	Residuals	11	59	5.36		
*Philodina spp.*	Competition	1	96	96	52.36	0.00194
	Residuals	4	7.33	1.83		
*P. tetraurelia*	Competition	1	14.4	14.4	1.075	0.33
	Residuals	8	107.2	13.4		

## Discussion

Colonization of species between patches in a metacommunity ultimately affects diversity and coexistence of species in the system. Studies of competition-colonization tradeoffs between species often categorize species by their (presumably fixed) relative colonization abilities and then use this to make predictions about coexistence [e.g. 10]. However, our results emphasize that movement between patches or habitats may vary with context in ways not immediately evident. Our original hypothesis was that good colonizers should elevate colonization rates in the presence of competition, perhaps because in the context of a competition-colonization tradeoff they are thought to rely on (and it is energetically favourable) a competition-avoiding, dispersal-based strategy. Conversely, we did not expect that species classified as strong competitors (i.e. poor colonizers) would increase their movement.

However, our results indicate that both species considered good competitors and good colonizers can (but do not necessarily) increase their colonization rates in the presence of competition. Further, given that these increases in emigration rates occurred when competition was high, and hence the intrinsic rate of increase was unlikely to be heightened, it appears that for some species competitive pressure results in higher speeds (or distances) of dispersal. This result suggests that although models of colonization and competition rarely consider that dispersal might change with context, changes in competitive intensity may in fact alter colonization in important ways. However, context was important only in the case of two species, with the remaining two not showing colonization differences in the presence of competitors. Whether or not such context dependent changes would alter the position of the species along a competition-colonization tradeoff or simply alter the magnitude of colonization rates is not clear. In addition, future studies should try to ascertain the mechanisms by which competition might influence dispersal.

Generally, for the protist species we used, it has been shown that there is a tendency for smaller species to increase population size faster, and colonize empty patches better because of both a numerical effect (more individuals to disperse) and a behavioral effect (they tend to move further and faster) [10], [24], [25]. *Colpidium*, one of the ‘colonizers’, showed a decrease in the time to colonize all patches in the metacommunity with one competitor, but not in the presence of multiple competitors (Fig. 2). In previous work with this species, smaller, faster moving individuals have been observed in multispecies assemblages, ostensibly due to a reduction in food quantity or quality or a selective effect (MWC, personal obs.). The increased colonization rates in *Philodina* are more difficult to explain. Previous work has concluded that while rotifers differ in various aspects of swimming (speed, timing, duration, etc.) they do not appear to change this behavior in response to declining food [26]. It may be that *Philodina* is responding to other stimuli, such as the accumulation of ciliate waste or associated changes in pH. While it is attractive to categorize species into classes such as ‘good colonizer’ or ‘good competitor’ or even as r- or K-selected species, most species are not so easily categorized [8]. Though dispersal is costly, the optimal strategy will always have some level of dispersal and the incorporation of both these strategies [6], [8]. Thus organisms may be more flexible than we often believe.

It is worth noting that the three different treatments (monocultures, 2 species microcosms, and four species microcosms) differed not just in the amount of intraspecific competition, but in terms of the total starting densities in the microcosm (500, 1260, and 2000 total individuals in 100 mL, respectively). However, given that these starting densities are very low compared to the final densities reached in the microcosms (microcosms with these species can reach densities of more than 10,000 individuals/mL –M. Cadotte, unpublished data), we would suggest that these minor differences in competitive pressure are negligible, compared to the difference in identity of interspecific competitors.

This study has some important limitations beyond the small number of species investigated. We did not directly measure the effects of competition on individuals or individual dispersal capabilities. Differences in density across treatments could have influenced intra- and interspecific competitive behavior, either on its own or in combination with other species interactions. Further, we looked at the effect of competition, though predation is often thought to influence prey behavioral responses and dispersal [27], though even these can be complex and difficult to predict [28]. However, *Blepharisma spp.* is an omnivore that can feed on other protists, with previous studies showing that *Colpidium* declines rapidly in the presence of *Blepharisma* [29] –though the length of our experiment was not long enough to see an accumulation of carnivore morphs in *Blepharisma*, but *Colpidium* may have perceived them as a predator. Extensions to this study could involve more species and species combinations. In conclusion, our results show that emigration rates, and therefore colonization success may be contingent on local biotic interactions. Predictions based on measures of dispersal should include species’ responses to the presence of competitors.
